# The evaluation of tactile dysfunction in the hand in type 1 diabetes: a novel method based on haptics

**DOI:** 10.1007/s00592-022-01903-1

**Published:** 2022-05-31

**Authors:** F. Picconi, C. P. Ryan, B. Russo, S. Ciotti, A. Pepe, M. Menduni, F. Lacquaniti, S. Frontoni, A. Moscatelli

**Affiliations:** 1 Unit of Endocrinology, Diabetes and Metabolism, S. Giovanni Calibita, Fatebenefratelli Hospital, Rome, Italy; 2 Department of Systems Medicine and Centre of Space Biomedicine, University of Rome Tor Vergata, Rome, Italy; 3 Laboratory of Neuromotor Physiology, IRCCS Fondazione Santa Lucia, Rome, Italy; 4 Department of Systems Medicine, University of Rome Tor Vergata, Rome, Italy; 5 Research Centre “E. Piaggio” and Department of Information Engineering, University of Pisa, Pisa, Italy; 6 Unit of Neurology, S. Giovanni Calibita Fatebenefratelli Hospital, Rome, Italy

**Keywords:** Diabetic neuropathies, Touch, Haptic technology, Motion perception, Vibration, Hand

## Abstract

**Aims:**

We present an innovative method based on haptics for the evaluation of the sense of touch in the hand, in people affected by type 1 diabetes.

**Methods:**

Forty individuals affected by diabetes and 20 healthy controls took part in the study; the diabetes group was further divided into two subgroups based on vibration sensitivity in the lower limb. By means of a novel haptic device, tactile sensitivity in the fingertip was measured as the ability of the participants to discriminate slip motion speed.

**Results:**

Tactile sensitivity was significantly lower in individuals affected by diabetes as compared to controls. Depending on the subgroup, the difference from the controls was equal to 0.11 (95% CI from 0.029 to 0.186) and to 0.267 (95% CI from 0.198 to 0.336). Within the diabetes group, tactile sensitivity correlated with vibration sensitivity in the upper (*p* = 0.001) and lower limb (*p* = 0.003). A significant relationship between nerve conduction parameters and tactile sensitivity was found (*p* = 0.03). Finally, we combined the different predictors (clinical, vibratory and electroneurography data) by using cluster analysis; tactile sensitivity was found to be significantly different between different clusters (*p* = 0.004).

**Conclusions:**

Early signs of tactile dysfunction in the hand were found in individuals affected by diabetes, even in absence of diabetic neuropathy. The protocol presented in this study is a promising tool for the assessment of tactile dysfunction in the hand in people affected by type 1 diabetes.

**Supplementary Information:**

The online version contains supplementary material available at 10.1007/s00592-022-01903-1.

## Introduction

Diabetic peripheral neuropathy is one of the most frequent and debilitating complications of diabetes mellitus. It is heterogeneous in nature and encompasses various degrees of sensory and motor dysfunctions [1]. The typical neuropathy is a symmetrical, length-dependent sensorimotor polyneuropathy attributable to metabolic and microvessel alterations as a result of chronic diabetes and cardiovascular risk covariates. As in other length-dependent neuropathies [2], it affects sensory fibres in the lower limbs, although it may also involve sensory fibres of the upper limbs in later stages of the disease. Since there are no currently approved disease-modifying therapies once the disease is fully manifested, early recognition of signs and symptoms is essential to prevent complications and improve quality of life [1].

According to current guidelines, diabetic peripheral neuropathy can be classified as possible, probable, subclinical or confirmed [1]. The diagnosis of possible and probable neuropathy is based on symptoms and signs which are typically measured by subjective neurological exam, 10 g Semmes–Weinstein monofilament test and vibration tests. Confirmed neuropathy requires both suggestive findings on nerve conduction exams and a symptom(s) or sign(s) of neuropathy. Standard quantitative sensation tests are used for the diagnosis of possible/probable neuropathy. These include light touch testing, 10 g Semmes–Weinstein monofilament test on the foot sole and vibration-based tests. Sensitivity to vibrations can be tested with the tuning fork or mechatronic devices like the Biothesiometer, and it is evaluated on the foot (hallux and malleolus). Different protocols have been developed to evaluate sensitivity to vibrations with stimuli ranging from 64 to 128 Hz, the latter of which is above the sensitivity range of slowly adapting fibres [3–6]. Recently, the VibroSense Metre was used to evaluate the vibrotactile sense of paediatric type 1 diabetes individuals in a broad frequency range [7].

These classical methods of evaluation are typically conducted in the lower limb. However, the hand is among the regions in our body with the highest tactile sensitivity [3]. Cutaneous stimuli from the hand are encoded by four types of afferent fibres, classified as slowly adapting (SA-I and SA-II) and fast adapting (FA-I and FA-II) and processed in specialised regions of the central nervous system [8–10]. Intact sensory feedback in the hand, in particular sensitivity to slip motion, is essential for dexterous manipulation of objects [10, 11]. For example, humans take advantage of partial slip to gauge the stability of a contact and react appropriately when slippage is about to happen [10]. Likewise, we slide our fingertips on objects to perceive some of their properties by touch, with stereotyped movements known as the exploratory procedures [12]. It is possible to study the role of slip motion in human touch by means of specialised robotic interfaces, known as haptic interfaces (haptics from Ancient Greek ἀπτ℩ϰός, meaning “related to touch” but also “able to touch” or “able to grasp”) [3]. By using haptic interfaces, it is possible to deliver motion stimuli on the skin with a high degree of precision [13–15]. Using this technology haptic interfaces, recent studies evaluated how different cues, such as spatiotemporal cues, high-frequency vibrations and gross deformation by shear force, are combined for the representation of slip motion by touch [16, 17].

Recently, smart quantitative sensation tests have been developed, which aim to increase the reproducibility of the tests [18]. Only a few studies developed methods for the evaluation of tactile sensitivity in the hand in people affected by diabetes [19–22]. In the discussion of this manuscript, we provide an overview of the recent studies on this topic. To the best of our knowledge, none of these methods specifically investigated slip motion. Because of the importance of slip motion in daily life, e.g. in dexterous manipulation and grasping, it is paramount to include it in the evaluation of individuals affected by diabetes.

## Methods

### Participants

A total of 40 people affected by type 1 diabetes (age: 37.7 ± 12, *mean* ± *sd*) and 20 healthy controls (age: 33.5 ± 10), age- and sex-matched to the people affected by diabetes, took part in the experiment. The testing procedures for the experiments were approved by the ethics committee of the Santa Lucia Foundation, in accordance with the guidelines of the Declaration of Helsinki for research involving human subjects. Informed written consent was obtained from all participants involved in the study. Inclusion criteria for the people affected by type 1 diabetes were (1) documented diagnosis of type 1 diabetes, according to ADA criteria; (2) age between 18 and 65 years; (3) treated with continuous subcutaneous insulin infusion or with multiple daily insulin injections and with 7-day continuous glucose monitoring (CGM); (4) HbA1c < 9.5% [23]. People affected by diabetes with a history of possible confounding diseases (central nervous system diseases, entrapment mononeuropathies, cervical or lumbosacral radiculopathies, alcohol abuse, vitamin deficiency, malignancy treated with chemotherapy agents) were excluded from the study.

Participants of the control group (median age equal to 30 years with interquartile range between 27.8–37.2; 9 females and 11 males) did not have a history of diabetes mellitus or any of the confounding pathologies mentioned above. They were evaluated by a senior physician to exclude pathological conditions that would interfere with the measurements.

### Clinical and electrophysiological measurements

People affected by type 1 diabetes underwent a general medical examination and an ophthalmological examination with fundus photography. Neurological evaluation included Michigan Neuropathy Screening Instrument (MNSI), 10 g Semmes–Weinstein monofilament test, vibratory perception threshold of upper and lower limbs by Biothesiometer (Meteda, San Benedetto del Tronto, Italy; stimulus frequency equal to 100 Hz) bilateral standard sensory motor nerve conduction studies of upper and lower limbs (Medtronic Keypoint EMG equipment, Skovlunde, Denmark). Nerve conduction was evaluated in the radial sensory nerve, sural nerve and peroneal nerve (control) of 34 individuals affected by diabetes. The velocity of conduction, amplitude and latency were measured. The measurements were taken from either the left or right side of the body, or both. When both left and right nerves were measured, the average value was computed for further analyses.

### Laboratory measurements

After an overnight fast, blood and urine samples were obtained for the determination of laboratory measurements. Plasma glucose concentrations were measured by the hexokinase method by a Modular P Analyzer (Roche, Basel, Switzerland). Glycated haemoglobin (HbA1c) was analysed by high-performance liquid chromatography by VARIANT 2 (BioRad Laboratories, Munich, Germany). Plasma total cholesterol, high-density lipoprotein cholesterol (HDL chol) and low-density lipoprotein cholesterol (LDL chol) were analysed with a colorimetric enzymatic method by CHOD-PAP (Roche, Basel, Switzerland). Plasma triglycerides were analysed with a colorimetric enzymatic method by GPO-PAP (Roche Diagnostics, Basal, Switzerland). Urinary albumin was determined by the Tina-quant immunoturbidimetric assay by Cobas (Roche Diagnostic, Indianapolis, USA) and urinary creatinine by an enzymatic colorimetric test (Beckmann Coulter, USA).

### Evaluation of tactile sensitivity with haptics

**Fig. 1:**
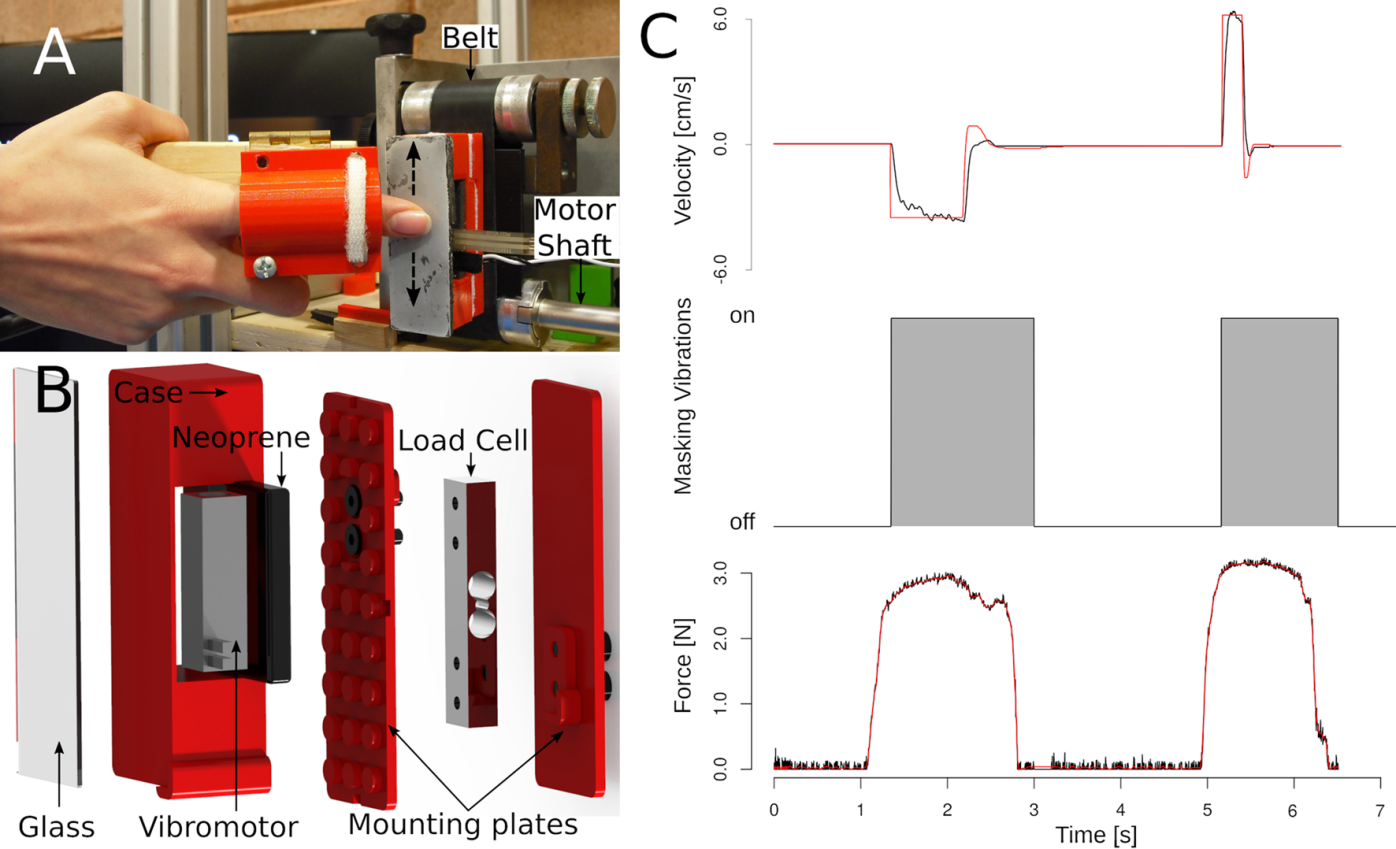
The Open Touch haptic device used for the study. **a** Participants contacted the movable plate of the device with their index finger. **b** Expanded image of the device including the vibromotor and the load cell. **c** Slip motion velocity, masking vibrations and normal force in the reference and comparison stimuli of a single trial

We developed a novel device that we called OpenTouch to evaluate tactile sensitivity to slip motion [24]. The apparatus controls the vertical displacement of a contact surface (microscope glass) either up or down (Fig. 1). The final position of the contact surface and its motion speed were precisely controlled by a servomotor (miniature drive system). To measure contact force, a force sensor (load cell) was placed between the box and the contact surface. Vibration stimuli were generated by a high-definition voice coil transducer controlled with a standard PC audiocard (HDA Intel PCH, Santa Clara, USA). Prior to the experiment, masking vibrations were recorded with an accelerometer to measure the amplitude and frequency of the signal. A comprehensive description of this apparatus can be found in the supplementary information.

### Stimuli and procedures

In the two diabetes affected groups, the blood glucose value was measured prior to the experiment session and it was corrected otherwise if it was not within the range of 80 and 140 mg/dl. Next, the 10 g Semmes–Weinstein monofilament test and Biothesiometer test were performed. Biothesiometer testing was performed on the left and right lower limbs and in the left and right upper limbs at the standard locations, i.e. malleolus and hallux for the lower limb and ulnar styloid prominences, radial styloid prominences and interphalangeal joints of the index finger, middle finger and thumb for the upper limb.

Testing procedure in the haptic test was the same for both individuals affected by diabetes and healthy participants. Participants sat on an office chair in front of the apparatus, resting their right index finger on the finger holder. A curtain hid the device from the participants’ sight. Throughout each experimental session, participants wore earplugs and headphones playing pink noise in order to mask external sounds. Each experimental session consisted of 120 trials and lasted approximately 30 min. Each trial included a reference and a comparison stimulus. The order of the two was counterbalanced across trials. The participants were instructed to push on the contact surface with the index finger to start the tactile stimulus. The servomotor and the voice coil were actuated when the normal force exceeded the threshold value of 1.5 N. The surface moved either upward or downward, with the motion direction of the second stimulus always being the opposite of the first one. The motion speed was equal to 3.4 cm/sec in the reference stimulus, and it was pseudo-randomly chosen between five values ranging from 0.6 cm/sec and 6.4 cm/sec in the comparison. After each trial, participants reported whether the surface moved faster in the first or in the second stimulus interval. The path length of the stimulus was pseudo-randomly chosen within a range of 1.0–1.4 cm. In half of the trials, masking vibrations consisting of a 100 Hz sinusoidal wave were delivered synchronously with the motion stimulus.

### Statistical analysis

The binary responses to the haptic test were analysed by means of general linear models (GLM) in each participant and generalised linear mixed models (GLMM) across all participants. The slope of the GLM/GLMM provides an estimate of tactile sensitivity [16, 25]. The higher the slope of the model, corresponding to a steeper response curve, the higher the tactile sensitivity. For examples of simulated tactile sensitivity as predicted by the slope of the psychometric function refer to supplementary figures 1 and 2. The difference in tactile sensitivity between controls and individuals affected by diabetes was assessed by means of dummy predictors in GLMM, and the difference in sensitivity was computed with the bootstrap method.

Principal component regression was used to test for the relationship between tactile sensitivity and sensitivity to vibrations assessed by Biothesiometer. Principal component analysis (PCA) was first performed on Biothesiometer data to summarise the original variables with a smaller number of orthogonal principal components (PC). We selected the first PC that accounted for more than 80% of variance. Then, a linear model (least squares linear regression) was used to predict tactile sensitivity by this PC. Principal component regression was used to test the relationship between nerve conduction PC and tactile sensitivity. By using cluster analysis (*k*-means clustering), we partitioned the group affected by diabetes based on age, disease duration, HbA1c, MNSI, Biothesiometer tests in the lower limb and nerve conduction in the sural nerve. Two clusters were selected based on the silhouette method [26]. Two-sample *t* tests were performed to determine if tactile sensitivity was significantly different between these two clusters. Finally, multiple linear regression models were used to test the relationship between tactile sensitivity and the following predictors: age, sex, masking vibrations, group affected by diabetes vs. control group (all participants), disease duration, MNSI and HbA1c (diabetes group only).

## Results

### Biothesiometer test

Demographic, clinical and laboratory data of the group affected by diabetes are reported in Table 1. People affected by diabetes were divided into two subgroups based on their sensitivity to vibrations measured with a Biothesiometer. They were classified as BIO1 if they had alterations on vibration sensitivity in at least two sites of stimulation in the lower limb (*n* = 20) and BIO0 otherwise (*n* = 20). None of the individuals affected by diabetes were positive for the 10 g Semmes–Weinstein monofilament test. By using ANOVA, we verified that the ages of the participants across the three groups (control, Bio0 and Bio1) did not differ significantly (*F* value = 0.898, *p* = 0.413). The average BMI in the Bio0 group was 23 (sd = 3.8) and the average BMI in the Bio1 group was 24.9 (sd = 4.8). We performed a *t* test to evaluate if the difference in BMI between the two groups was statistically significant. Within the diabetic group there was not a significant difference in BMI between the Bio0 and Bio1 groups (*t* = − 1.2341, *df* = 26.639, *p*-value = 0.228). The Biothesiometer values for each area tested are shown in supplementary table 1.

**Table 1:** Demographic data and laboratory measurements in participants affected by diabetes

Demographic data			
	Total No. 40	Bio0 No. 20	Bio1 No. 20
Age (yrs)	37.0 (28.0–45.5)	37.0 (27.8–43.5)	36.0 (28.0–47.0)
Sex (Female)	24 (60.0%)	13 (65.0%)	11 (55.0%)
Disease duration (yrs)	18.0 (5.8–31.2)	18.5 (4.8–25.0)	18.0 (11.8–32.0)
HbA1C (%)	7.6 (6.8–8.4)	7.4 (6.8–8.0)	8.0 (7.2–8.8)
Triglycerides (mg/dl)	56.5 (46.5–79.0)	69.5 (51.8–83.2)	50.0 (45.0–62.2)
Total cholesterol (mg/dl)	178.5 (164.5–200.2)	174.5 (166.0–182.2)	191.0 (151.8–203.5)
HDL (mg/dl)	54.0 (50.0–64.2)	54.5 (51.5–66.2)	53.5 (49.8–57.2)
LDL (mg/dl)	106.7 (95.0–126.5)	100.6 (93.3–107.3)	114.8 (101.8–140.9)
Microalbuminuria (mg/mmol)	1.0 (1.0–2.0)	1.0 (0.8–1.5)	1.0 (1.0–2.0)
Participant count of abnormal MNSI	23 (59.0%)	6 (30.0%)	17 (89.5%)
Peripheral neuropathy (%)	11 (32.4%)	4 (23.5%)	7 (41.2%)

### Tactile sensitivity

**Fig. 2:**
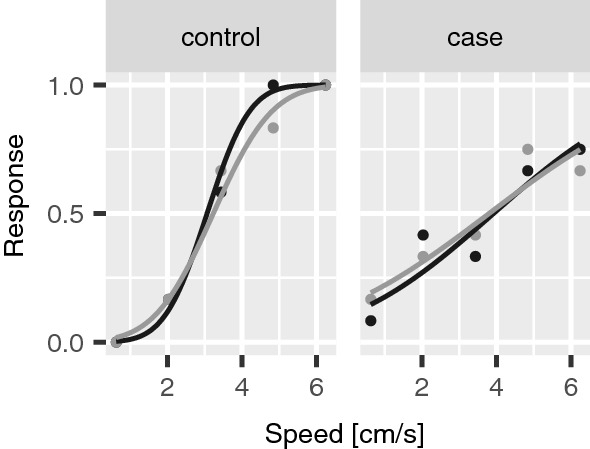
Tactile sensitivity in the haptic test. The response in the haptic test in a control (left panel) and in an individual affected by diabetes (right panel). Non-masking vibration condition is shown in black and masking vibration condition is shown in grey. In the control participant, to the left, the curves are steeper (i.e., the slope parameter is higher) as compared to the case, to the right. This means that tactile sensitivity to slip motion was higher in the control than in the case (Color figure online)

**Fig. 3:**
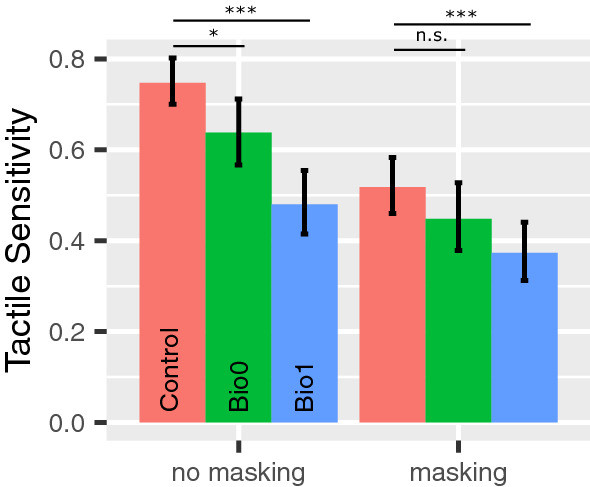
Barplots of the slopes of GLM (tactile sensitivity) in control (red), BIO0 (green) and BIO1 (blue) in non-masking and masking vibration conditions. The error bars represent the 95% confidence intervals (CI) computed with the bootstrap method

Figure 2 shows the responses of two representative participants (one healthy control and one person affected by diabetes) in the haptic test, in the two experimental conditions with and without masking vibrations. The steepness of the curve (slope) is a measurement of participants’ tactile sensitivity to slip motion. As shown in the figure, the slope was higher in the control as compared to the person affected by diabetes. Figure 3 shows the difference in tactile sensitivity (slope) between groups estimated with GLMM. Without masking vibrations, tactile sensitivity was significantly lower in BIO0 as compared to controls (difference = 0.11, 95% CI from 0.029 to 0.186). It was also significantly lower in BIO1 as compared to controls in the two experimental conditions, with masking vibrations (difference = 0.145, 95% CI from 0.063 to 0.223) and without masking vibrations (difference = 0.267, 95% CI from 0.198 to 0.336). Masking vibration significantly impaired tactile sensitivity in the three groups (average difference = − 0.165, 95% CI from − 0.207 to − 0.122), in accordance with our previous study on healthy participants [24]. The average tactile sensitivity values are reported in supplementary table 2.

### Biothesiometer test and tactile sensitivity

**Fig. 4:**
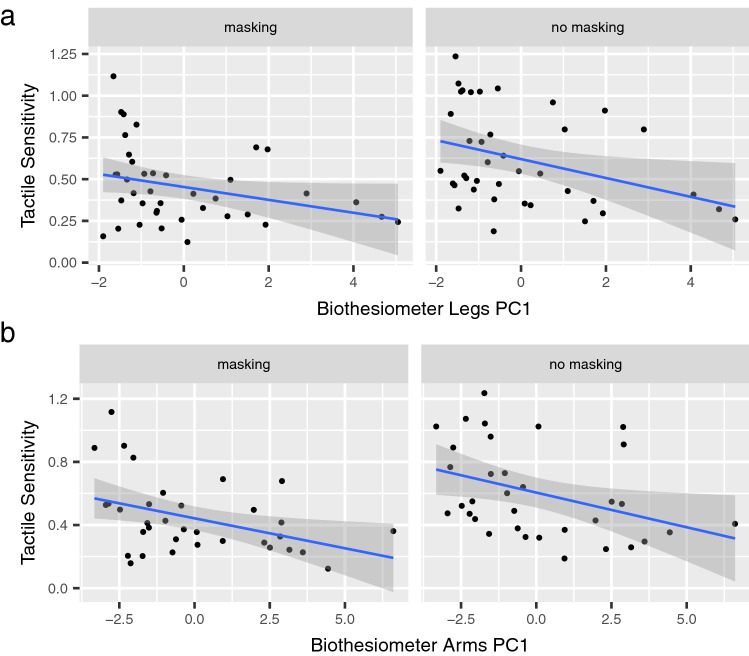
Sensitivity in the haptic test and Biothesiometer. **a** Linear regressions of tactile sensitivity and PC1 of Biothesiometer of the legs in masking vibration and non-masking vibration conditions. Tactile sensitivity was estimated by the slope of the GLM as explained in the text. **b** Linear regressions of tactile sensitivity and PC1 of Biothesiometer of the arms in masking vibration and non-masking vibration conditions

Next, we used principal component regression to test the linear relationship between tactile sensitivity and Biothesiometer test in the lower limbs. The first component (PC1) accounted for more than 80% of the variance of Biothesiometer data and therefore it was included as a single predictor in the linear model. We found a significant negative relationship between tactile sensitivity and PC1 of the lower limb in both non-masking and masking vibration conditions (*t*-value = − 3.064, *p* = 0.003). Similarly, we observed a negative relationship between tactile sensitivity and PC1 of the upper limb, in both non-masking and masking vibration conditions (*t*-value = − 3.401, *p* = 0.001). The linear relationship in the lower and in the upper limb is illustrated in Fig. 4. Overall, tactile sensitivity was lower in individuals affected by diabetes with a lower performance on the Biothesiometer test, in the upper and the lower limbs. Tactile sensitivity was also correlated with PCA of a combination of the upper and lower limb Biothesiometer results (refer to supplementary figure 5).

### Nerve conduction test and tactile sensitivity

Of the 34 individuals affected by diabetes that underwent the electroneurographic examination, 11 were classified as neuropathic by an expert neurologist. In two individuals affected by diabetes the amplitude of the radial nerve was lower than the threshold values reported in the literature (refer to supplementary figure 3) [27].

**Fig. 5:**
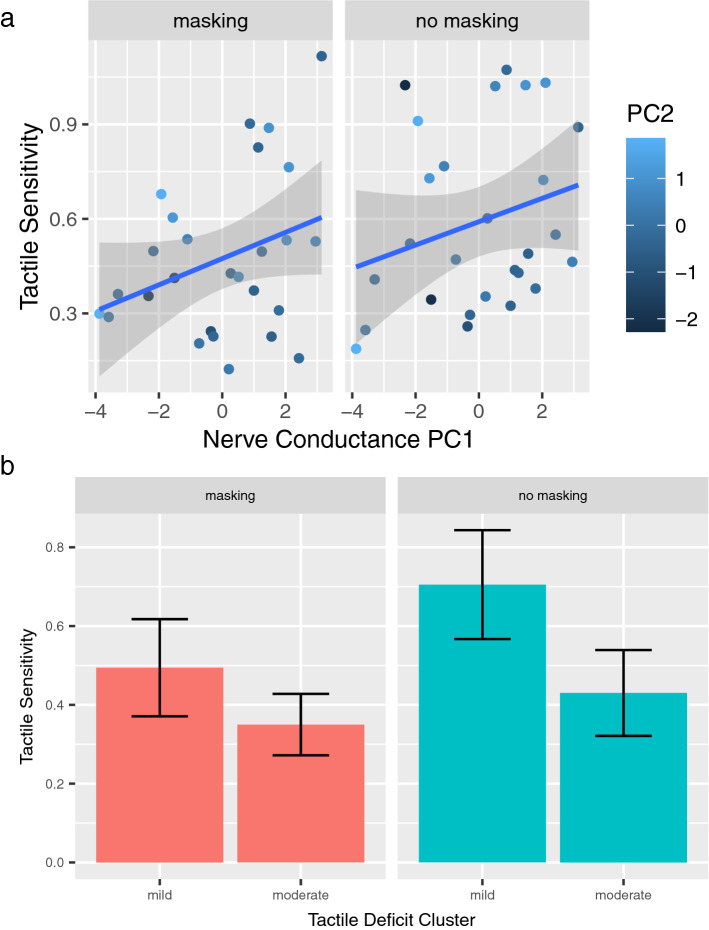
Sensitivity in the haptic test and nerve conductance. **a** Linear regressions of tactile sensitivity and principal components of nerve conductance. PC1 is shown on the *x*-axis and PC2 is scaled in blue. Tactile sensitivity was estimated by the slope of the GLM as explained in the test. **b** Bar plots of the tactile sensitivity of the two clusters (mild and moderate) in masking (left panel) and non-masking vibration conditions. The error bars represent the 95% confidence intervals (CI) computed with the bootstrap method

We used principal component regression to test the relationship between tactile sensitivity to slip motion and nerve conduction parameters of the radial and sural sensory nerves. The first two PCs of the principal component analysis (PCA) accounted for more than 80% of the variance (refer to supplementary figure 4). The first component accounts for dysfunction in nerve conduction. Individuals affected by diabetes with high scores on the first component have high velocity and amplitude of conduction and lower latency, that is, they had absent or lowered nerve dysfunction in the two nerves. The second component represents the difference between the two nerves. A positive score in the second component is associated with lower or absent radial nerve dysfunction and a sural nerve dysfunction, and vice versa a negative score indicates that the dysfunction is mostly associated with the radial nerve. Using a linear model, we regressed the first two principal components onto tactile sensitivity to slip motion. There was a positive relationship between PC1 and tactile sensitivity that was statistically significant (*t*-value = 2.231, *p* = 0.03). This means that people affected by diabetes with lower or absent nerve dysfunction have higher tactile sensitivity whereas those affected by diabetes with higher nerve dysfunction have a lower tactile sensitivity. PC2 was not significantly associated with tactile sensitivity (*t*-value = 1.522, *p* = 0.134). Figure 5a shows the nerve conduction PCs against tactile sensitivity in both non-masking vibration conditions and masking vibration conditions. Nerve conductance data of the sural and the radial nerve are reported in the supplementary tables 3 and 4, respectively.

### Cluster analysis

Cluster analysis was used to combine multiple collinear variables that are typically tested in clinical settings. The *k*-means clustering algorithm divided the group affected by diabetes into two clusters based on the following variables: age, disease duration, HbA1C, MNSI, Biothesiometer test in the lower limbs and electrophysiological parameters (velocity of conductance, amplitude and latency in the sural nerve). We named the two clusters as “mild” and “moderate” tactile deficit (Fig. 5b). Individuals affected by diabetes in “mild” cluster have lower MNSI scores compared to the “moderate” (the centroid being 1.4 in the first cluster compared to 2.9 in the second), lower Biothesiometer score (ranging from 10.8 to 12.2 across different sites in “mild” vs 12.9 to 16.9 in “moderate”), higher amplitude in the sural nerve (18 vs 9) and shorter disease duration (7.8 years vs 33.1 years). HbA1C was slightly less in the “mild” group (7.2 vs 8.1). Finally, individuals affected by diabetes in the “mild” group were on average 20 years younger with respect to the “moderate” group. Two-sample *t* tests were conducted to determine if tactile sensitivity was different between these two clusters and this difference was significant in non-masking vibration conditions (*t*-value = 3.127, *p* = 0.004). There was also a nonsignificant trend in masking vibration conditions (*t*-value = 1.979, *p* = 0.058).

### Demographic data and tactile sensitivity

Using a multiple linear regression model, we tested the relationship between tactile sensitivity and the following predictors: age, sex, participant group (individuals affected by diabetes or controls) and masking vibration condition (with or without masking). Both age and sex affected tactile sensitivity. We found a negative relationship between tactile sensitivity and age (*t*-value = − 2.411, *p* = 0.017) and significant effect of sex, with male participants performing worse than females (*t*-value = − 2.198, *p* = 0.03). In accordance with previous studies [16] the presence of masking vibrations significantly reduced tactile sensitivity (*t*-value = − 5.018, *p* < 0.001). Tactile sensitivity was significantly lower in the individuals affected by diabetes as compared to the controls (*t*-value = − 3.108, *p* = 0.002).

Finally, we used linear regression models to evaluate the effect of the following clinical and demographic variables on tactile sensitivity: disease duration, MNSI and HbA1c. We found a negative relationship between tactile sensitivity and disease duration (*t*-value = − 2.472, *p* = 0.016). This means that the people affected by diabetes with a longer disease duration had lower tactile sensitivity. Then we tested the relationship between tactile sensitivity and MNSI. There was a negative relationship between these two variables albeit nonsignificant (*t*-value = − 1.445, *p* = 0.153). The effect of HbA1c on tactile sensitivity was not significant (*t*-value = − 0.983, *p* = 0.329).

## Discussion

In this study, we developed a non-invasive test for the evaluation of sensitivity to slip motion in the hand in individuals affected by type 1 diabetes. Tactile stimuli were delivered with a custom-made haptic interface including a miniature drive system that ensured high precision and reliability of the motion stimuli. Sensitivity to slip motion was evaluated with our interface in individuals with diabetes that either tested positive or tested negative to the Biothesiometer and in an equal number of healthy controls. As in our previous study on healthy individuals [16], we evaluated the sensitivity to slip motion in two experimental conditions, with and without masking vibrations. Additionally, we tested tactile dysfunction in the upper and lower limb by means of standard methods including the Biothesiometer and 10 g Semmes–Weinstein monofilament test, MNSI and electrophysiological measurements.

Both invasive (nerve conduction test) and non-invasive standard exams were found to be significantly related to tactile sensitivity in the hand as measured by our method. Tactile sensitivity as measured by the novel haptic device was significantly lower in both groups affected by diabetes (Bio0 and Bio1) as compared to controls. Tactile sensitivity significantly correlated with Biothesiometer testing both in the upper and the lower limb. Tactile sensitivity also significantly correlated with nerve conductance. People affected by diabetes with lower or absent nerve dysfunction had higher tactile sensitivity whereas those affected by diabetes with higher nerve dysfunction had a lower tactile sensitivity. Tactile sensitivity evaluation by our protocol is based on perceptual judgement by the participants which can be affected by many factors at multiple sites of the nervous system including mechanoreceptors, nerve fibres and myelin and its sensory processing in the CNS. Nerve conduction study instead evaluates a part of this system namely the propagation of the nervous signal along the nerve fibre and it was used in our study for standard neurological evaluation of the patients. Although we found a significant correlation between tactile sensitivity (perceptual judgement) and nerve measurements, factors like peripheral and central sensory noise may have affected the actual strength of correlation. In the future it may be interesting to compare the tactile sensitivity measured in our protocol with other measurements such as cutaneous biopsy [1] and evaluation of the mechanoreceptors [28] and the small nerve fibres [29, 30]. Overall, our results suggest that subtle alterations in tactile sensitivity in the hand may occur also in the early course of the disease.

Other recent studies looked to evaluate tactile sensitivity in the hand. One study investigated the detection of object shape in a population of blind individuals with diabetes, blind people without diabetes and blindfolded controls [19]. A similar test exploited tactile exploration of object curvature as a method for evaluation of tactile function [20]. A third study measured tactile detection of bumps that varied in height on a smooth surface and correlated the outcomes with Meissner corpuscle density [21]. While these studies included hand evaluation, they required an examiner to present the stimulus and record the responses. The detection of texture by touch was evaluated in an automatic manner by the ARDITA device [22]. It is composed of a tactile pins-array scale with stimuli delivered to the right index finger. Although this device provides test reproducibility, none of the aforementioned studies investigated slip motion, as in our study.

Slip motion stimuli, like the ones used in our test, produce complex patterns of skin deformation that recruits both slowly and fast adapting fibres [31]. Sensitivity to slip motion in the hand is of the utmost importance for dexterous manipulation of objects [10, 32] and for the control of hand movements in grasping and in reaching tasks [33, 34]. Accordingly, individuals affected by diabetes with and without diabetic peripheral neuropathy present a lower safety margin in their grip force while holding an object, and neuropathic individuals also present an impairment in tests of finger dexterity (e.g. nine-hole peg test) [35, 36]. Both a reduced sensitivity to slip motion and the loss of the motor units [37] may concur to this impaired dexterity. In future studies, it will be possible to use our method to correlate manual dexterity and sensitivity to slip motion in individuals affected by diabetes.

Strengths and limitations of the study. Limitations of the study are the following, which can all be addressed in future work. The nerve conduction studies were conducted in a subgroup of DM participants (*n* = 34). Metabolic characterisation was performed in the DM group only; the control group did not perform a metabolic evaluation, but were carefully examined and selected based on thorough medical history. We did not test the median nerve because we limited our investigation to the nerves included in the standard nerve conduction evaluation. The response of the median nerve may have a stronger association with tactile sensitivity because it innervates the index fingertip. In accordance with our previous study [16], delivering high-frequency vibrations (masking vibrations) to the fingertips of participants reduced their ability to discriminate motion speed by touch. This is consistent with recent findings in the neuroscience of touch, showing the complex interplay between motion speed and vibrations at both mechanical and neural level [38, 39]. Despite its importance for basic science studies, the use of masking vibrations during the haptic test did not provide a clear advantage for the classification of the individuals affected by diabetes. The difference in tactile sensitivity of individuals affected by diabetes with respect to the controls (GLMM), and between individuals affected by diabetes with mild and moderate disease (cluster analysis), was higher without masking vibrations. Therefore, it will be possible to reduce the duration of the testing procedure by including only trials without masking vibrations. The demographic variables of age and gender affected tactile sensitivity in both individuals affected by diabetes and controls. In the future, it will be possible to extend the study to a larger cohort of participants, for a more precise characterisation of the different variables. Individuals affected by diabetes were screened for carpal tunnel syndrome (CTS) by amnestic report and neurological examination. However, considering the high prevalence of CTS among individuals affected by diabetes, we cannot exclude that this was misdiagnosed in some of the patients [40, 41].

Because peripheral diabetic neuropathy is a length-dependent neuropathy, it typically affects first sensory fibres in the lower limbs and sensory fibres of the upper limbs in later stages of the disease. The aim of the present study was to investigate the damage to the upper limb which is generally overlooked in the literature. In future studies it will be important to extend this protocol to the lower limbs, for an early evaluation of the disease. Testing the foot will also overcome the confounding factor of CTS. A device for testing tactile sensitivity in the foot is currently under development by our research group.

The novel test presented in this study can provide an important tool for the assessment of tactile sensitivity in the hand in individuals affected by diabetes. This method of testing is non-invasive and cost-effective. The evaluation of sensitivity to slip motion may be important for the early detection, diagnosis and monitoring of tactile dysfunction in the hand, which our findings suggest may occur also in an early stage of the disease. The new haptic test may provide an important tool for the evaluation of tactile sensitivity in the hand in type 1 diabetes.

## Prior presentation

Parts of this study were presented at the 31st Annual Meeting of NEURODIAB 2021, at the 30th Annual Meeting of NEURODIAB 2020, at the 29th Annual Meeting of NEURODIAB 2019, at the 57th virtual EASD Annual Meeting 2021 as an oral presentation and at the 56th virtual EASD Annual Meeting 2020 as a poster presentation.

## Supplementary Information

Below is the link to the electronic supplementary material.

Supplementary file1 (PDF 5 kb)

Supplementary file2 (PDF 5 kb)

Supplementary file3 (PDF 15 kb)

Supplementary file4 (PDF 7 kb)

Supplementary file5 (PDF 13 kb)

Supplementary file6 (DOCX 10 kb)

Supplementary file7 (DOCX 7 kb)

Supplementary file8 (DOCX 6 kb)

Supplementary file9 (DOCX 7 kb)
