# Supplementary figures and images for: The evaluation of tactile dysfunction in the hand in type 1 diabetes: a novel method based on haptics
Source: Acta Diabetol. 2022 May 31;59(8):1073–82. doi: 10.1007/s00592-022-01903-1 (PMC9242965; doi:10.1007/s00592-022-01903-1)

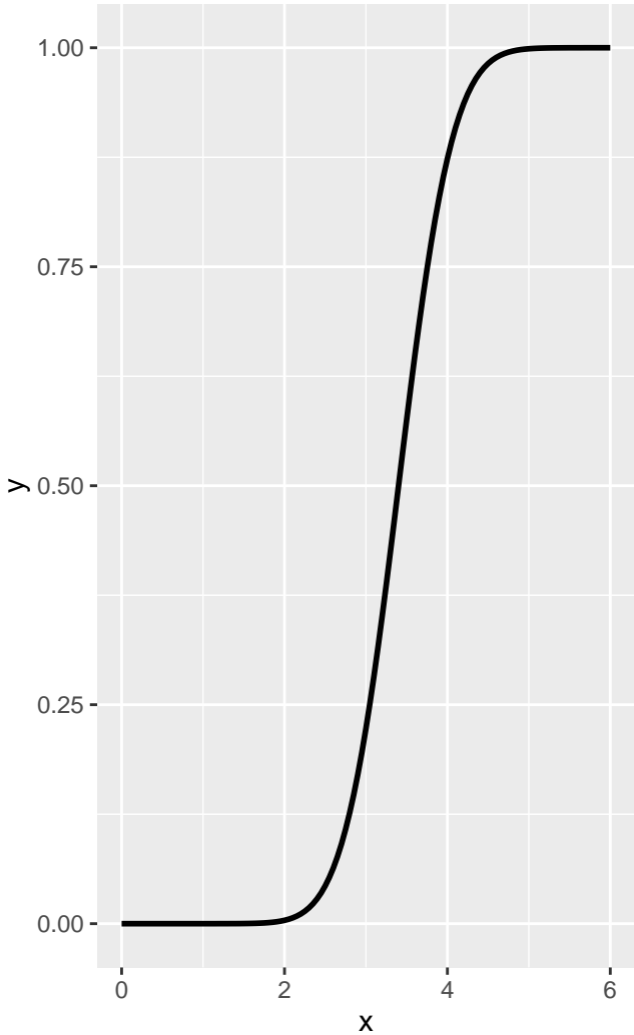

Supplement: Supplementary file 1 — Supplementary file1 (PDF 5 kb) [file 592_2022_1903_MOESM1_ESM.pdf]

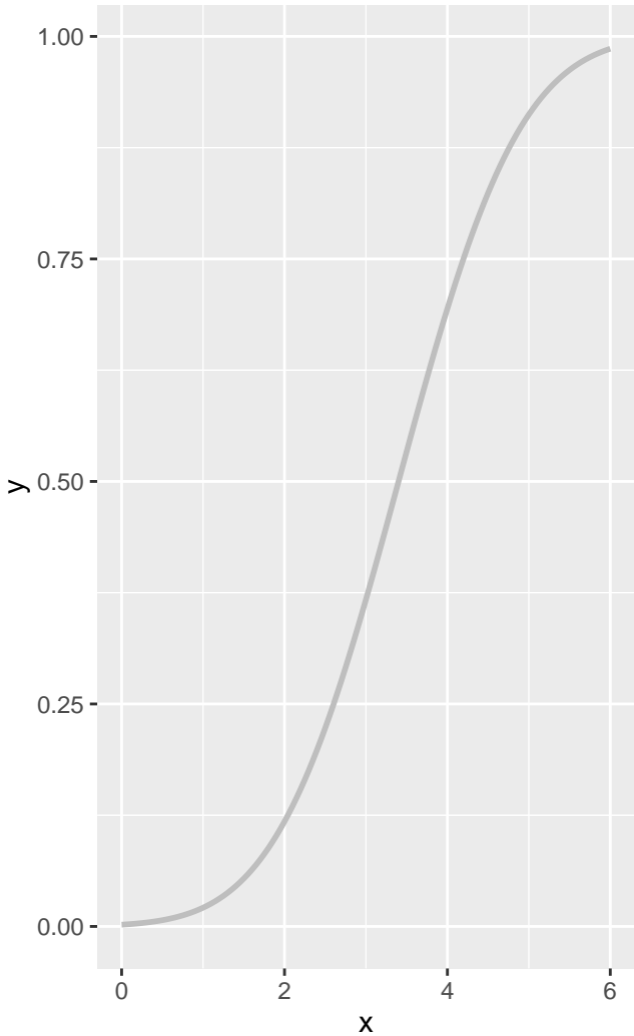

Supplement: Supplementary file 2 — Supplementary file2 (PDF 5 kb) [file 592_2022_1903_MOESM2_ESM.pdf]

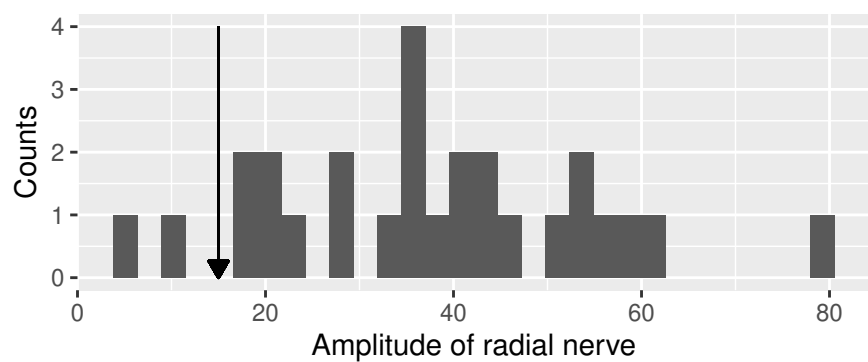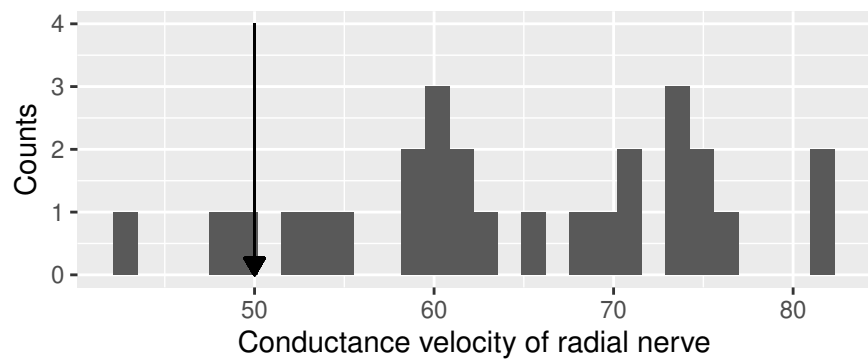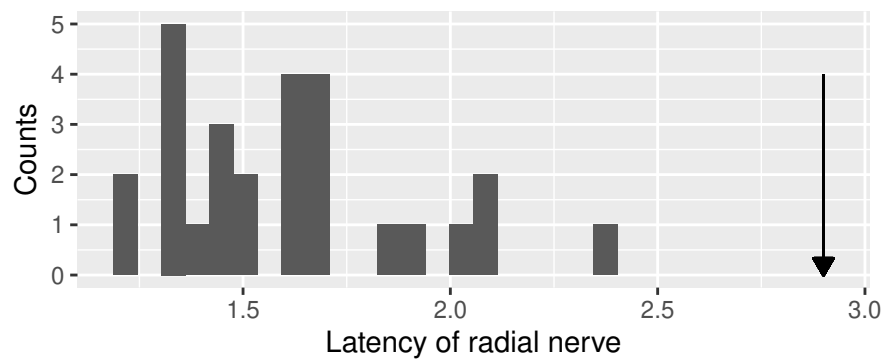

Supplement: Supplementary file 3 — Supplementary file3 (PDF 15 kb) [file 592_2022_1903_MOESM3_ESM.pdf]

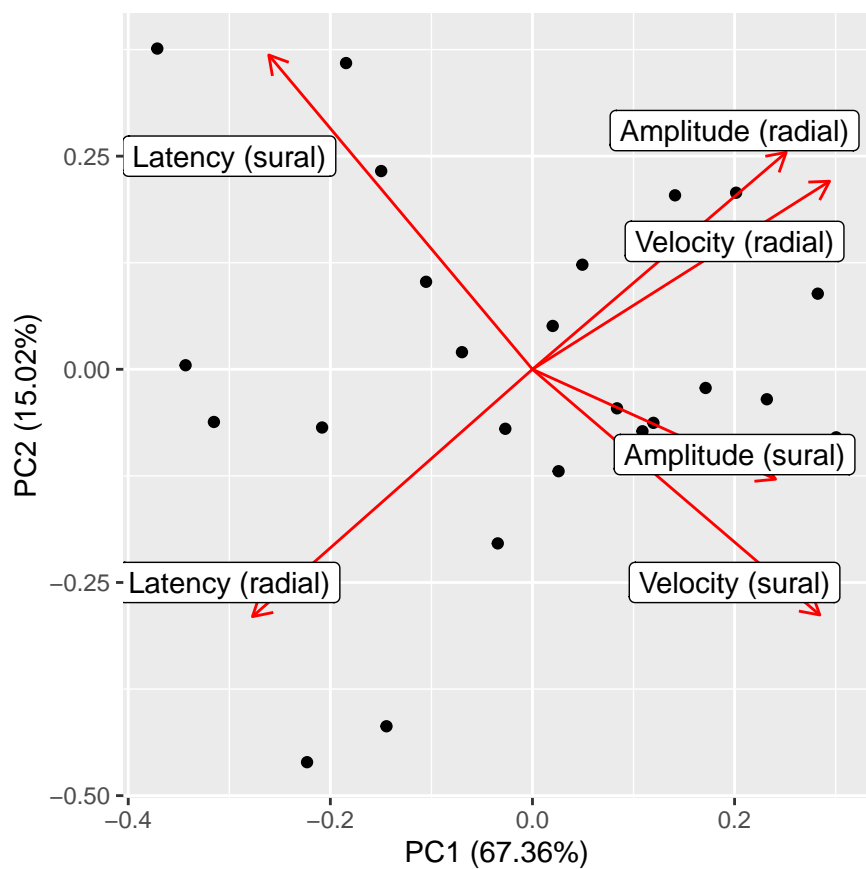

Supplement: Supplementary file 4 — Supplementary file4 (PDF 7 kb) [file 592_2022_1903_MOESM4_ESM.pdf]

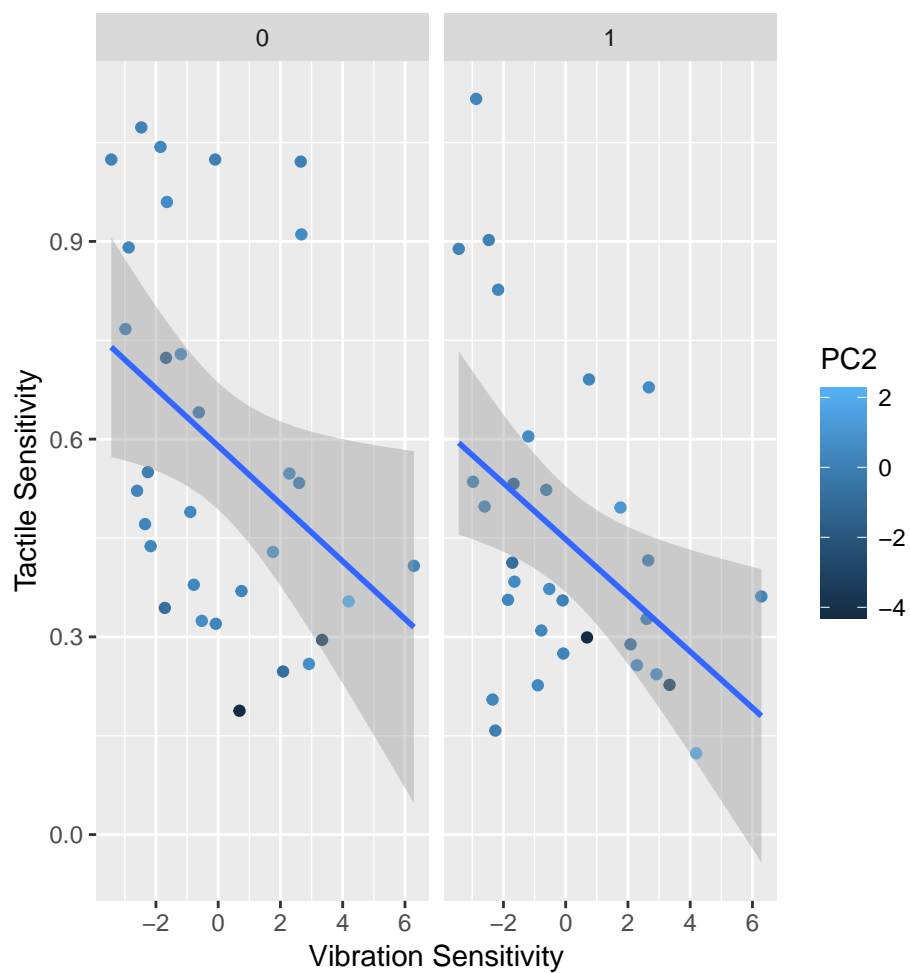

Supplement: Supplementary file 5 — Supplementary file5 (PDF 13 kb) [file 592_2022_1903_MOESM5_ESM.pdf]
