# Supplementary material for: The evaluation of tactile dysfunction in the hand in type 1 diabetes: a novel method based on haptics
Source: Acta Diabetol. 2022 May 31;59(8):1073–82. doi: 10.1007/s00592-022-01903-1 (PMC9242965; doi:10.1007/s00592-022-01903-1)
Supplement: Supplementary file 6 — Supplementary file6 (DOCX 10 kb) [file 592_2022_1903_MOESM6_ESM.docx]

## **Supplementary Information**

### **The Open Touch Haptic Device**

The Open Touch consists of a motion device that we used to displace the contact surface up and down at the required speed. Two rollers with a rigid plane in between rotated a plastic belt (270 mm length x 30 mm width). The two rollers were vertically aligned, and the lower roller was connected via a metal shaft to a Faulhaber motion system. The motion system was composed of a DC-Micromotor (Faulhaber 3242G024CR combined with 7:1 Gearheads 32A), a high-resolution encoder (Faulhaber IE3-512), and a position and speed controller (Faulhaber MCDC3006SRS). A rigid plastic box having a cuboid shape (75 x 20 x 20 mm) was attached to the belt and a contact surface consisting of a smooth microscope glass (75 x 25 x 1.5 mm) was attached to the other side. A vibromotor Haptuator Mark II by TactileLabs, connected to a MAX9744 audio amplifier by Adafruit, was placed in a small niche inside the box to generate masking vibrations. Vibration stimuli were generated by a standard PC audio card (HDA Intel PCH). To detect contact, a load cell CZL616C Phidgets Inc. was placed between the box and a basement firmly attached to the belt. In this manner, the rotation of the motor produced a vertical displacement of the box. The load cell was connected to an Arduino Uno board converting voltage values to force measurements. The load cell interconnected the box with the basement through mounting plates providing interlocking attachments. A custom Matlab code controlled the masking vibration, the contact force measurements, and the motion stimuli. A one degree-of-freedom finger holder was attached to the frame of the setup, supporting the participant’s finger. Before the experiment phase, the load cell was calibrated through a set of calibration weights, ranging from 0.98 to 4.91 N. The masking vibrations were retrieved in amplitude and frequency with an accelerometer attached to the contact plate and analyzed offline. Masking vibrations were recorded through an Arduino Uno board commanded with a Matlab script.

### **Tactile sensitivity and sensitivity to high-frequency vibrations in the upper and lower limb**

Principal component regression was used to test the relationship between tactile sensitivity and combined vibration sensitivity of the upper and lower limb (supplementary figure 3). The first two components accounted for 67% of the variance. The first principal component accounted for the correlation of tactile sensitivity and biothesiometer data while the second principal component separated the upper and lower biothesiometer data. We then regressed tactile sensitivity on PC1 and PC2 using the least squared model. There was a significant negative relationship between tactile sensitivity and PC1 in both masking and non-masking vibration conditions (t = -3.55, p < 0.001). There was a non-significant negative relationship between tactile sensitivity and PC2 in both masking and non-masking vibration conditions (t = -0.004, p = 1.0). This model predicts that patients with a higher threshold value on biothesiometer (both upper and lower limbs) have a lower tactile sensitivity on the haptic test.

Supplementary table captions

1. Biothesiometer results in patient groups. The columns include the total, Bio0 and Bio1 group. Values indicate the mean and standard deviation.
2. Tactile sensitivity measured by the haptic test. The columns include the total participants, control group, Bio0 and Bio1 groups in the no masking and masking vibration conditions. The values reflect the mean and standard deviation.
3. Nerve conduction results of the sural and radial nerves. The first column includes all participants affected by diabetes followed by the Bio0 and the Bio1 groups. The tables provide the mean and standard deviation values for the amplitude, conductance velocity and latency in each nerve.

Supplementary figure captions

1. Simulated data of a single participant: a psychometric function is used to fit the response during the haptic test. The x-axis is the actual speed of the stimuli whereas the y-axis is the percentage of perceiving the comparison stimulus as faster. The slope of the psychometric function is a measurement of tactile sensitivity. This figure represents an individual with high tactile sensitivity where the slope is steep.
2. Simulated data of a single participant. The x-axis is the actual speed of the stimuli whereas the y-axis is the percentage of perceiving the comparison stimulus as faster. This is an example of an individual with low tactile sensitivity where the slope is shallow.
3. Histogram illustrating the results of the nerve conduction studies across participants affected by diabetes. The three nerve conduction parameters are included on the x-axes of each histogram. The y-axes show the number of patients who scored in the indicated nerve conduction value. The black arrows illustrate the thresholds for physiological values in healthy individuals.
4. Biplot of the first two principal components (PC) of the nerve conduction studies. Nerve conduction parameters are highlighted on the plot. The individual dots represent participants affected by diabetes.
5. Vibration sensitivity and tactile sensitivity. Tactile sensitivity was linearly regressed on vibration sensitivity illustrated on the x-axes. The vibration sensitivity is the principal component (PC1) of the combined upper and lower limb biothesiometer results. PC2 is illustrated in color code. The no masking vibration condition (0) is shown in the left panel and the masking vibration condition (1) is shown in the right panel.
