# Supplementary material for: The evaluation of tactile dysfunction in the hand in type 1 diabetes: a novel method based on haptics
Source: Acta Diabetol. 2022 May 31;59(8):1073–82. doi: 10.1007/s00592-022-01903-1 (PMC9242965; doi:10.1007/s00592-022-01903-1)
Supplement: Supplementary file 7 — Supplementary file7 (DOCX 7 kb) [file 592_2022_1903_MOESM7_ESM.docx]

| Supplementary Table 1: Biothesiometer Results in Diabetes Mellitus groups (Mean ± Standard Deviation) | | | | |
| --- | --- | --- | --- | --- |
|  | **Total** |  | **Bio0** | **Bio1** |
| Malleolus (Right) | 12.51 (±5.68) |  | 9.29 (±1.49) | 15.57 (±6.49) |
| Malleolus (Left) | 12.31 (±6.68) |  | 9.03 (±2.05) | 15.43 (±8.02) |
| Thumb (Right) | 15.13 (±7.11) |  | 10.08 (±2.00) | 19.93 (±6.90) |
| Thumb (Left) | 15.27 (±7.97) |  | 9.95 (±1.81) | 20.32 (±8.29) |
| Ulnar (Right) | 10.50 (±4.50) |  | 8.89 (±2.29) | 12.11 (±5.56) |
| Ulnar (Left) | 10.36 (±2.87) |  | 8.86 (±1.88) | 11.86 (±2.94) |
| Radial (Right) | 10.26 (±3.21) |  | 9.25 (±2.71) | 11.28 (±3.41) |
| Radial (Left) | 10.14 (±2.98) |  | 8.92 (±1.93) | 11.36 (±3.38) |
| Index Finger (Right) | 9.43 (±3.05) |  | 7.86 (±1.39) | 11.00 (±3.46) |
| Index Finger (Left) | 8.62 (±1.99) |  | 7.81 (±1.09) | 9.44 (±2.36) |
| Middle Finger (Right) | 9.88 (±3.26) |  | 8.25 (±1.54) | 11.50 (±3.73) |
| Middle Finger (Left) | 9.37 (±2.65) |  | 8.06 (±1.35) | 10.68 (±3.00) |
| Ring Finger (Right) | 10.10 (±3.70) |  | 8.09 (±1.62) | 12.12 (±4.12) |
| Ring Finger (Left) | 10.50 (±3.44) |  | 8.76 (±1.86) | 12.24 (±3.82) |
