# Supplementary material for: The evaluation of tactile dysfunction in the hand in type 1 diabetes: a novel method based on haptics
Source: Acta Diabetol. 2022 May 31;59(8):1073–82. doi: 10.1007/s00592-022-01903-1 (PMC9242965; doi:10.1007/s00592-022-01903-1)
Supplement: Supplementary file 8 — Supplementary file8 (DOCX 6 kb) [file 592_2022_1903_MOESM8_ESM.docx]

| Supplementary Table 2: Tactile Sensitivity Across all Participants divided by group (Mean ± Standard Deviation) | | | | |
| --- | --- | --- | --- | --- |
|  | **Total**  **No. 60** | **Ctr**  **No. 20** | **Bio0**  **No. 20** | **Bio1**  **No. 20** |
| no masking | 0.70 (±0.29) | 0.83 (±0.25) | 0.71 (±0.27) | 0.55 (±0.28) |
| masking | 0.48 (±0.23) | 0.55 (±0.20) | 0.50 (±0.27) | 0.39 (±0.17) |
