# Supplementary material for: The evaluation of tactile dysfunction in the hand in type 1 diabetes: a novel method based on haptics
Source: Acta Diabetol. 2022 May 31;59(8):1073–82. doi: 10.1007/s00592-022-01903-1 (PMC9242965; doi:10.1007/s00592-022-01903-1)
Supplement: Supplementary file 9 — Supplementary file9 (DOCX 7 kb) [file 592_2022_1903_MOESM9_ESM.docx]

| Supplementary Table 3: Nerve Conductance Data of the Sural Nerve (Mean ± Standard Deviation) | | | |
| --- | --- | --- | --- |
|  | **Total** | **Bio0** | **Bio1** |
| Amplitude (microvolts) | 13.6 (±8.2) | 15.3 (±8.8) | 11.7 (±7.2) |
| Conduction velocity (m/s) | 46.1 (±7.3) | 47.3 (±6.2) | 44.8 (±8.5) |
| Latency (milliseconds) | 3.2 (±0.8) | 3.0 (±0.5) | 3.4 (±1.1) |

| Supplementary Table 4: Nerve Conductance Data of the Radial Nerve (Mean ± Standard Deviation) | | | |
| --- | --- | --- | --- |
|  | **Total** | **Bio0** | **Bio1** |
| Amplitude (microvolts) | 37.5 (±17.0) | 42.2 (±18.8) | 31.5 (±12.9) |
| Conduction velocity (m/s) | 64.5 (±10.3) | 65.2 (±9.6) | 63.6 (±11.5) |
| Latency (milliseconds) | 1.6 (±0.3) | 1.6 (±0.2) | 1.7 (±0.4) |
